# Prevalence of Drug-resistant *Klebsiella pneumoniae* in Iran: A Review Article

**Published:** 2018-03

**Authors:** Mohsen HEIDARY, Mohammad Javad NASIRI, Hossein DABIRI, Samira TARASHI

**Affiliations:** 1. Dept. of Microbiology, School of Medicine, Iran University of Medical Sciences, Tehran, Iran; 2. Dept. of Microbiology, School of Medicine, Shahid Beheshti University of Medical Sciences, Tehran, Iran; 3. Microbiology Research Center (MRC), Pasteur Institute of Iran, Tehran, Iran

**Keywords:** *Klebsiella pneumoniae*, Drug resistance, Iran

## Abstract

**Background:**

The infections caused by drug resistant strains of *Klebsiella pneumoniae* are becoming an important health problem worldwide. There are several reports on antimicrobial resistant status of *K. pneumoniae* in Iran. However, a comprehensive analysis on drug-resistant *K. pneumoniae* from different parts of Iran has not yet been performed.

**Methods:**

The searches were done according to several English and Persian databases including PubMed, Scopus, Iranmedex, and SID to identify studies addressing antibiotic resistant *K. pneumoniae* in Iran from Jan 1998 to Nov 2014. Comprehensive Meta-Analysis (V2.2, Biostat) software was used to analyze the data.

**Results:**

The incidence rate of imipenem and ceftazidime resistance in *K. pneumoniae* isolates was 3.2% (95% confidence interval [CI], 1.5–6.5) and 55.7% (95% CI, 46.9–64.1), respectively. The highest rate of resistance in isolates of *K. pneumoniae* was seen against ampicillin (82.2%), aztreonam (55.4%) and nitrofurantoin (54.5%).

**Conclusion:**

There is a relatively high prevalence of drug resistant *K. pneumoniae* isolates in Iran. Thus, a high degree of awareness among physicians and microbiologists, active infection control committee, appropriate antimicrobial therapy, improvement of hygiene condition and monitoring of drug resistant isolates are urgently needed in order to better control the emergence and spread of drug-resistant *K. pneumoniae* isolates in hospital settings.

## Introduction

*Klebsiella pneumoniae* is an important causative agent of hospital-acquired infections, including severe pneumonia, urinary tract infection as well as septicemia and wound infections (1, 2). This bacterium can survive in hospitals, persist on environmental surface and colonize different parts of human body. Therefore, transmission of this opportunistic pathogen can easily occur among patients via the hands of healthcare personnel. Furthermore, the increased use of antibiotics and persistent exposure of *K. pneumoniae* to a number of antimicrobial agents, facilitating the emergence of multidrug-resistant strains, which has further intensified the infection control strategies in many health care settings (3).

The most important resistant isolates of *K. pneumoniae* are carbapenem and cephalosporin resistant strains (4). These strains can cause serious infections in immunocompromised patients, in association with prolonged hospital, stays, limited therapeutic options and increased mortality rates, ranging from 12% to as high as 72%, depending on the study population (5–9). In these regards, a reliable estimate of the extent of drug resistant isolates of *K. pneumoniae* is needed for the programmatic management of drug resistant strains within the context of national infection control programs.

This study was designed to determine the prevalence of drug resistant strains of *K. pneumoniae* in Iran according to the Preferred Reporting Items for Systematic Reviews and Meta-Analyses statement (10, 11).

## Methods

### Search strategies

We conducted the search using PubMed, Web of Science, Cochrane library and Scopus for all studies addressing the prevalence of drug resistant strains of *K. pneumoniae* in Iran, from Jan 1998 to Nov 2014. The applied keywords include *Klebsiella*, *Klebsiella pneumoniae*, antibiotic resistance, antibiotic susceptibility, and Iran. Iranian databases including Iranmedex and Scientific Information Database (SID) were also searched (with Persian keywords).

### Inclusion and exclusion criteria

We considered all the original articles about the incidence rate of drug resistant strains of *K. pneumoniae* from hospital-acquired infections in Iran. These articles should reference to the standard method, which recommended by clinical and laboratory standards Institute (CLSI) for drug susceptibility testing of *K. pneumoniae* against; carbapenems, cephalosporins and the other most used antimicrobial agents. Due to the following reasons, some studies were excluded from our analysis. Articles have focused only on community acquired *K. pneumonia*e or focused only on *non-K. pneuomoniae* stains, and studies not used CLSI recommended drug susceptibility testing methods. Furthermore, case reports, meta-analyses or systematic reviews, letters to editor, review articles, non-English or Persian studies, and duplicate publication, were also excluded.

### Data extraction and definitions

The extracted data in current study include the first author’s name, the publication time, year of study, number of samples, and prevalence of drug resistant strains of *K. pneumoniae*. Two authors extracted data from all of the included studies independently and a third investigator reviewed results.

### Statistical analysis

The comprehensive meta-analysis software (ver. 2.0) was used to analyse the data. Because of the heterogeneity between studies, random effects models were used and tested with the Cochrane Q test. Moreover, Egger weighted regression and Begg rank correlation tests were performed to assess possible publication bias.

## Results

**Fig. 1:**
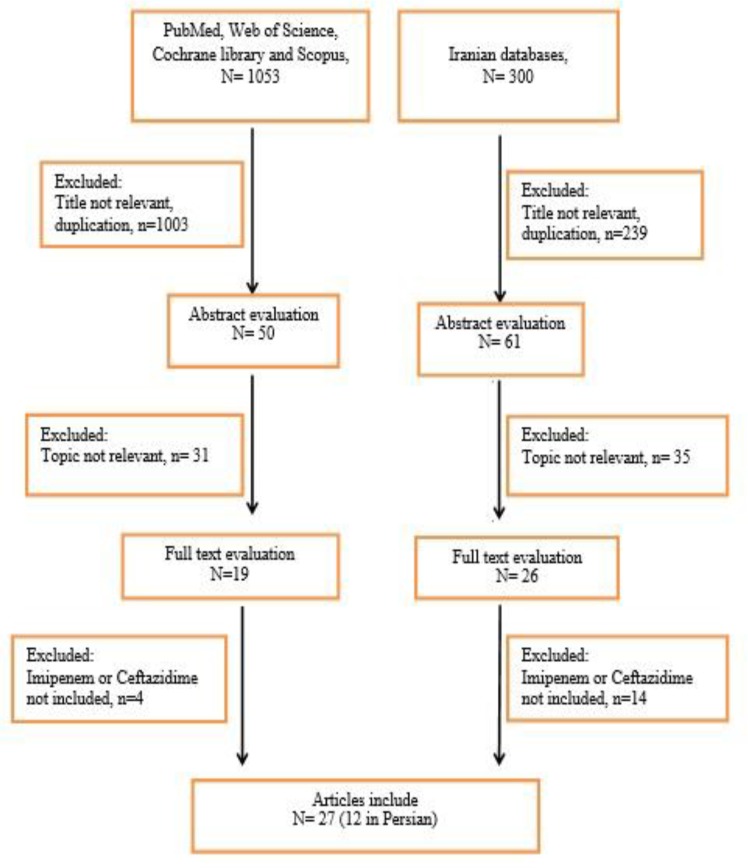
Summary of the literature search and study selection

**Fig. 2:**
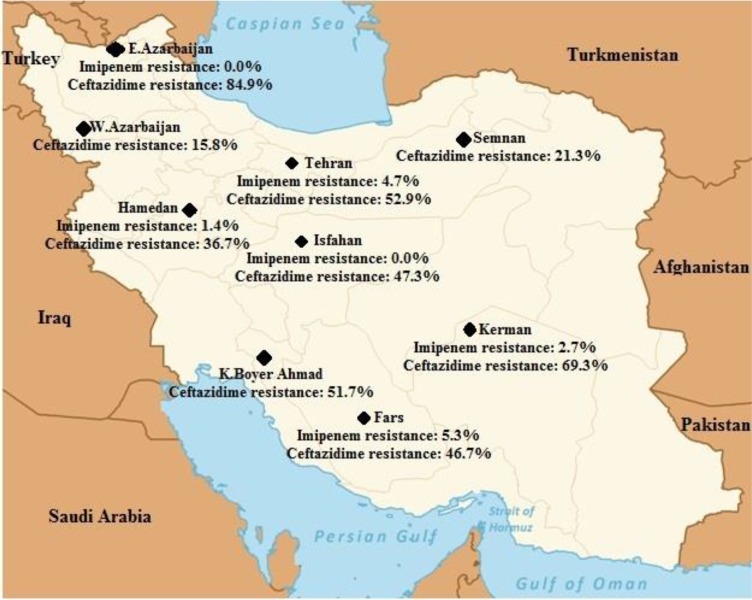
Distribution of drug-resistant *Klebsiella pneumoniae* in different regions of Iran

**Fig. 3:**
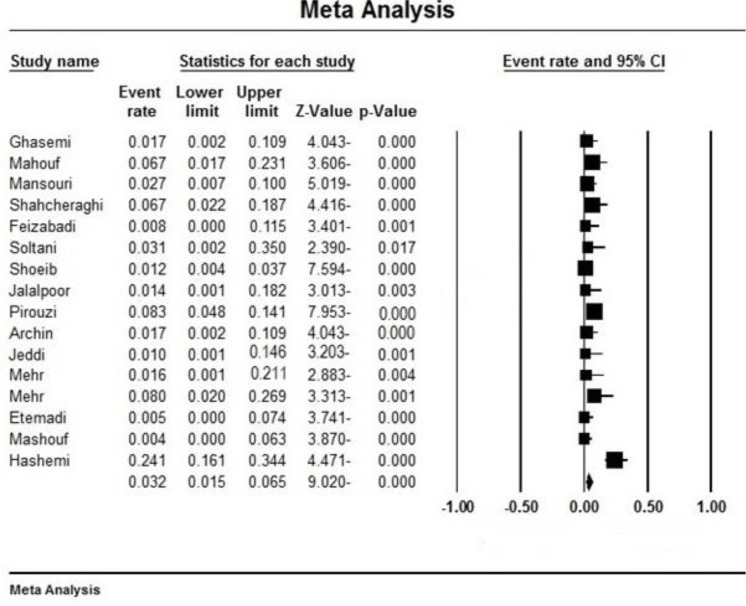
Forest plot of the meta-analysis on imipenem resistance. CI, confidence interval

**Fig. 4:**
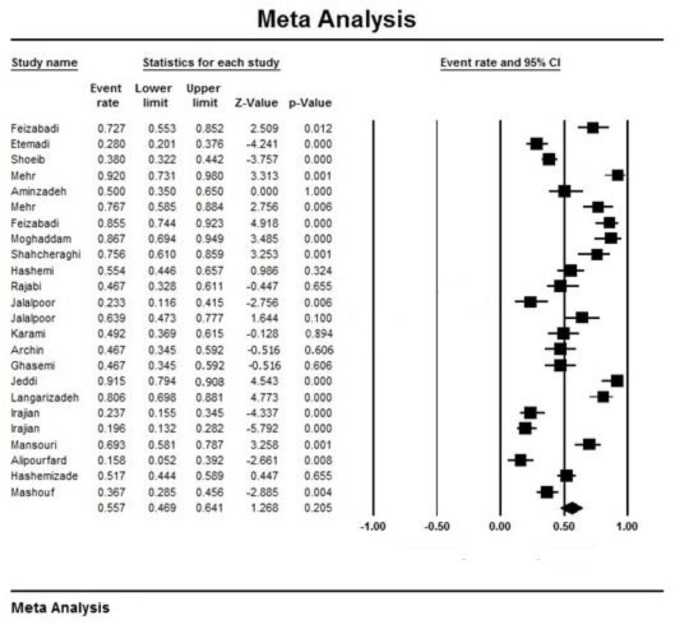
Forest plot of the meta-analysis on ceftazidime resistance. CI, confidence interval

Initially, 1353 articles were collected (Fig. 1). However, in a secondary screening, 1308 of them were excluded according to duplication, title, and abstract evaluation, and full-text of 45 papers was evaluated. Finally, 27 articles describing the prevalence of the ceftazidime- and/or imipenem-resistant strains of *K. pneumoniae* were selected for meta-analysis (Table 1). In all included studies, antimicrobial susceptibility testing had been performed using disc diffusion method as recommended by CLSI guidelines. Most of the studies were done in Tehran (n=11) compared with Isfahan (n=4), Fars (n=3), East Azerbaijan (n=2), Semnan (n=2), Hamadan (n=2), K. Boyer Ahmad (n=1), West Azerbaijan (n=1) and Kerman (n=1). Fig. 2 shows the distribution of drug-resistant strains of *K. pneumoniae* in different regions of Iran. The prevalence of imipenem and ceftazidime resistance was found to be 3.2% (95% CI, 1.5–6.5) and 55.7% (95% CI, 46.9–64.1), respectively (Table 2). Fig. 3 and 4 show the forest plot of the Meta-analysis of imipenem and ceftazidime resistant *K. pneumoniae*.

**Table 1:** Included studies after full-text evaluation

***References***	***Published time***	***Enrollment time***	***Province***	***Total number of samples***	***Isolates of Klebsiella penomoniae***	***Number of Ceftazidime* (%)**	***Resistance to Imipenem* (%)**
12	2007	2002–2005	Tehran	200	33	24(73)	-
13	2005	2003–2004	Tehran	115	100	28(28)	0(0)
14	2011	2006–2009	Tehran	250	250	95(38)	3(1)
15	2010	2007–2008	Tehran	101	25	23(92)	2(8)
16	2008	2007–2008	Tehran	164	40	20(50)	-
17	2009	2007–2008	Tehran	65	30	23(77)	0(0%)
18	2010	2008–2009	Tehran	81	62	53(85)	0(0%)
19	2014	2009–2010	Tehran	50	30	26(87)	-
20	2013	2009–2011	Tehran	360	45	34(76)	3(7)
21	2014	2011–2012	Tehran	83	83	46(55)	20(24)
22	2012	2011–2012	Tehran	120	45	21(47)	-
23	2011	2009–2010	Isfahan	211	30	7(23)	-
24	2014	2013–2014	Isfahan	123	15	-	0(0)
25	2011	2009–2010	Isfahan	167	36	23(64)	0(0)
26	2013	2010–2011	Isfahan	61	61	30(49)	-
27	2013	2009–2010	Fars	571	60	28(47)	1(2)
28	2012	2009–2010	Fars	328	144	-	12(8)
29	2013	2009–2010	Fars	60	60	28(47)	1(2)
30	2008	2007–2008	East Azarbaijan	88	47	43(91)	0(0)
31	2010	2008–2009	East Azarbaijan	72	72	58(81)	-
32	2010	2007–2008	Semnan	310	76	18(24)	-
33	2009	2007–2008	Semnan	382	107	21(20)	-
34	2014	2007–2008	Kerman	413	75	52(69)	2(3)
35	2005	1999–2001	West Azarbaijan	251	19	3(16)	-
36	2013	2010–2012	Kohgiluyeh and Boyer Ahmad	202	180	93(52)	-
**37**	2013	2011–2012	Hamedan	120	120	44(37)	0(0)
**38**	2009	2004–2006	Hamedan	209	30	-	2(7)

**Table 2:** The prevalence of imipenem and ceftazidime resistance among *Klebsiella pneumoniae*

***Subgroups***	***No. of study***	***Prevalence of drug resistance (95% CI)***	n/N*	***Heterogeneity Test***		***Egger’s test for publication bias***	
				***I^2^ (%)***	***P*-value**	***t***	***P*-value**
Overall effects of resistant to imipenem	16	3.2 (1.5–6.5)	46/1182	75.9	<.001	5.1	0.00016
Overall effects of resistant to ceftazidime	24	55.7 (46.9–64.1)	841/1686	92.2	<.001	2.4	0.02454

CI, confidence interval; n, number of events (drug resistance); N, total number of *Klebsiella pneumoniae* from the included studies

**Fig. 5:**
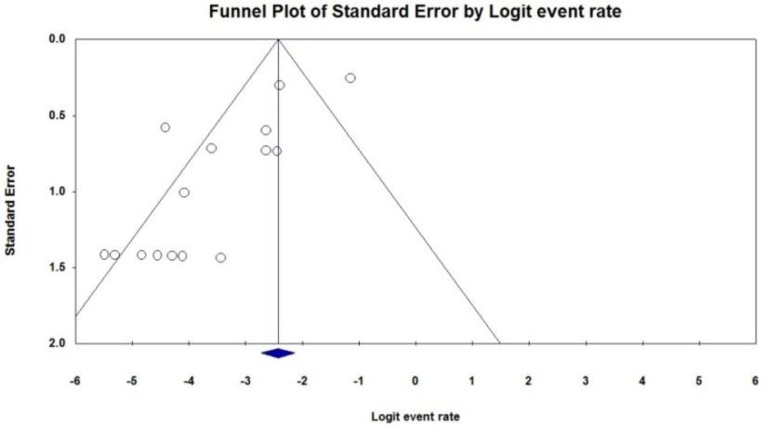
Funnel plot of the meta-analysis on imipenem resistance

**Fig. 6:**
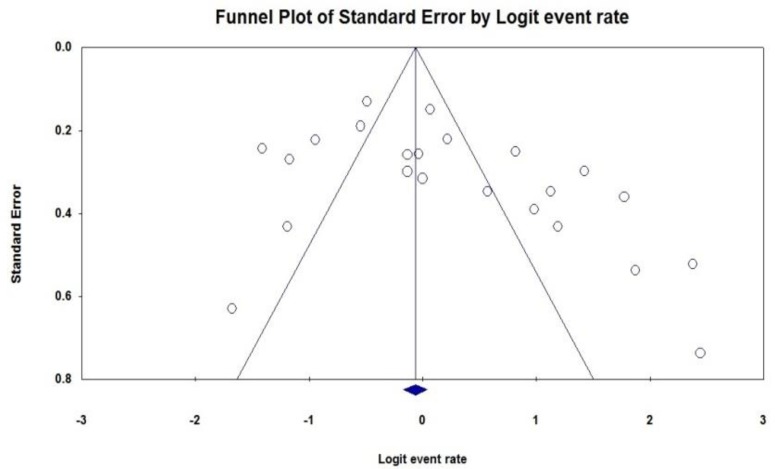
Funnel plot of the meta-analysis on ceftazidime resistance

Some evidence for publication bias for imipenem and ceftazidime was observed (*P*<0.05 for Begg rank correlation analysis; *P*<0.05 for Egger weighted regression analysis) (Fig. 5, 6). The resistance of *K. pneumoniae* to other important antimicrobial agents is shown in Table 3.

**Table 3:** Drug resistance status in *Klebsiella pneomoniae*

***References***	***Enrollment time***	***Case number***	***Carbapenem***		***Cephalosporins***				***Aminogly-cosides***		***Fluoroquinolones***	***Monobactam***	***Penicillins***	***Macrolid***	***Cotrimoxazole***
			**IMP^1^**	**MEM^2^**	**CAZ^3^**	**CTX^4^**	**CRO^5^**	**CPM^6^**	**AMK^7^**	**GM^8^**	**CIP^9^**	**AZT^10^**	**AMP^11^**	**NF^12^**	**TMP/SXT^13^**
**12**	2002–2005	33	-	-	24	19	19	-	14	21	15	26	-	14	-
**13**	2003–2004	100	0	-	28	-	20	-	9	30	20	-	-	31	-
**14**	2006–2009	250	3	-	95	91	86	100	53	82	85	-	-	-	-
**15**	2007–2008	25	2	-	23	22	23	22	24	-	17	-	24	13	18
**16**	2007–2008	40	-	-	20	-	19	-	8	14	12	-	40	16	20
**17**	2007–2008	30	0	-	23	6	20	25	20	16	18	-	30	18	18
**18**	2008–2009	62	0	-	53	56	47	44	14	30	32	59	-	16	47
**19**	2009–2010	30	-	-	26	25	-	-	16	17	26	-	30	-	-
**20**	2009–2011	45	3	13	34	37	-	33	11	-	32	32	-	-	38
**21**	2011–2012	83	20	20	46	50	49	30	12	29	46	49	65	-	-
**22**	2011–2012	45	-	-	21	-	-	-	-	-	43	-	-	23	31
**23**	2009–2010	30	-	-	7	5	-	-	0	7	6	-	21	10	8
**24**	2013–2014	15	0	0	-	15	-	15	8	-	12	-	15	10	-
**25**	2009–2010	36	0	-	23	21	-	22	12	-	13	-	33	7	28
**26**	2010–2011	61	-	-	30	49	37	-	-	-	-	-	-	-	-
**27**	2009–2010	60	1	-	28	34	-	29	5	8	13	19	60	-	26
**28**	2009–2010	144	12	-	-	-	-	-	61	65	42	-	23	-	43
**29**	2009–2010	60	1	-	28	34	-	29	5	8	13	19	60	-	26
**30**	2007–2008	47	0	-	43	42	44	39	5	-	-	41	-	-	-
**31**	2008–2009	72	-	-	58	-	-	-	31	53	31	-	-	68	69
**32**	2007–2008	76	-	-	18	19	-	-	-	19	35	-	73	-	41
**33**	2007–2008	107	-	-	21	24	-	-	-	19	21	-	97	-	27
**34**	2007–2008	75	2	-	52	25	-	27	-	48	21	-	-	-	35
**35**	1999–2001	19	-	-	3	-	-	-	0	5	3	-	14	-	3
**36**	2010–2012	180	-	41	93	87	81	-	40	65	31	83	-	138	108
**37**	2011–2012	120	0	-	44	50	52	30	-	32	20	52	-	-	49
**38**	2004–2006	30	2	-	-	-	3	3	11	13	7	19	60	-	-
**Mean**	-	-	46	74	841	711	500	448	359	581	613	399	645	364	635
**Rate**			(3.2)	(18.9)	(55.7)	(49.9)	(47.1)	(47.8)	(25.8)	(36.3)	(34.8)	(55.4)	(82.2)	(54.5)	(51.8)

**Abbreviations**: 1. IMP, imipenem; 2. MEM, meropenem; 3.CAZ, ceftazidime; 4. CTX, cefotaxime; 5. CRO, ceftrixone; 6. CPM, cefepime; 7. AMK, amikacin; 8. GM, gentamycin; 9.CIP, ciprofloxacin; 10. AZT, aztreonam; 11. AMP, ampicillin; 12. NF, nitrofurantoin; 13.SXT/TMP, trimethoprim/sulfamethoxazole

## Discussion

The emergence and spread of carbapenem and cephalosporin resistant strains of *K. pneumoniae* are a considerable threat to public health (2). The major goal of this systematic review was to evaluate the current situation and distribution of drug-resistant *K. pneumoniae* in Iran.

This analysis showed that 3.2% *K. pneumoniae* isolates from Iran was resistant to imipenem and 55.7% to ceftazidime. Thereby despite ceftazidime, the imipenem remains as a powerful weapon against *K. pneumoniae* isolates in Iran. In the current study more than half of *K. pneumoniae* isolates were resistant to other important antimicrobial agents such as aztreonam (55.4 %), nitrofurantoin (54.5%) and cotrimoxazole (51.8%), we highly recommend that antimicrobial test should be performed prior to any antibiotic prescription in *K. pneumonia* infections. Very low number of *K. pneumonia* population (17.8%) were sensitive to ampicillin suggesting ampicillin is not effective drug for empiric treatment of *K. pneumonia infections* unless we use it in combination with other relevant drugs.

The relatively high rates of drug resistant isolates of *K. pneumoniae* observed in this study may have several negative effects on public health issues (39). For example, this could cause difficulty in treating *K. pneumoniae* associated infections since fewer effective drugs are available for treating those highly drug-resistant strains. Unfortunately, these microorganisms are even showing rising rates of resistance to new expensive antibiotics subsequently considered the treatment of choice (40). This is due to the widespread use of broad-spectrum antibiotics in health care settings for empiric treatment of infections. Furthermore, patients infected with these pathogens require prolonged antimicrobial therapy that has considerable implications for the individual patient and for the health care settings. Finally, infections due to these highly resistant strains are reported to be associated with higher morbidity and mortality rates (41). In Iran, 50000 people die each year because of multidrug-resistant bacterial infections and that this costs Iranian economy 2.5 million dollars annually (4).

Some important reasons for the increasing rates of drug resistant isolates in Iran include limited infection surveillance programs, the lack of communication between physicians and microbiologists, lack of standardized or accepted criteria to determine drug resistant isolates, limited laboratory facilities, and poor sanitation. Therefore, active infection control committee, appropriate antimicrobial therapy, and improvement of hygiene condition will prevent or lower the emergence of antimicrobial-resistant pathogens (42).

Current review was carried out according to provinces of Iran and the published time. Because of many hospitals and health care centers in Tehran Province, Iran, patients from other provinces come to Tehran for better treatment. Therefore, most of the studies in this analysis belonged to Tehran, where the ceftazidime- and/or imipenem-resistant strains of *K. pneumoniae* mostly reported by researchers.

## Conclusion

There is a relatively high prevalence of drug resistant *K. pneumoniae* isolates in Iran. Thus, a high degree of awareness among physicians and microbiologists, active infection control committee, appropriate antimicrobial therapy, improvement of hygiene condition and monitoring of drug resistant isolates are urgently needed in order to better control the emergence and spread of drug-resistant *K. pneumoniae* isolates in hospital settings.

## Ethical considerations

Ethical issues (Including plagiarism, informed consent, misconduct, data fabrication and/or falsification, double publication and/or submission, redundancy, etc.) have been completely observed by the authors.
